# Pathological Role of Peptidyl-Prolyl Isomerase Pin1 in the Disruption of Synaptic Plasticity in Alzheimer's Disease

**DOI:** 10.1155/2017/3270725

**Published:** 2017-03-26

**Authors:** Lingyan Xu, Zhiyun Ren, Frances E. Chow, Richard Tsai, Tongzheng Liu, Flavio Rizzolio, Silvia Boffo, Yungen Xu, Shaohui Huang, Carol F. Lippa, Yuesong Gong

**Affiliations:** ^1^Jiangsu Key Laboratory for Functional Substance of Chinese Medicine, Department of Biopharmaceutics and Food Science, School of Pharmacy, Nanjing University of Chinese Medicine, Nanjing 210023, China; ^2^Department of Neurology, Drexel University College of Medicine, Philadelphia, PA 19102, USA; ^3^Department of Oncology, Mayo Clinic, Rochester, MN 55905, USA; ^4^Sbarro Institute for Cancer Research and Molecular Medicine, Center for Biotechnology, Temple University, Philadelphia, PA 19122, USA; ^5^Department of Molecular Science and Nanosystems, Ca' Foscari Università di Venezia, Via Torino 155, 30172 Venezia-Mestre, Italy; ^6^Department of Medicinal Chemistry, China Pharmaceutical University, Nanjing 21009, China; ^7^Department of Physiology, University of Pennsylvania School of Medicine, Philadelphia, PA 19104, USA

## Abstract

Synaptic loss is the structural basis for memory impairment in Alzheimer's disease (AD). While the underlying pathological mechanism remains elusive, it is known that misfolded proteins accumulate as *β*-amyloid (A*β*) plaques and hyperphosphorylated Tau tangles decades before the onset of clinical disease. The loss of Pin1 facilitates the formation of these misfolded proteins in AD. Pin1 protein controls cell-cycle progression and determines the fate of proteins by the ubiquitin proteasome system. The activity of the ubiquitin proteasome system directly affects the functional and structural plasticity of the synapse. We localized Pin1 to dendritic rafts and postsynaptic density (PSD) and found the pathological loss of Pin1 within the synapses of AD brain cortical tissues. The loss of Pin1 activity may alter the ubiquitin-regulated modification of PSD proteins and decrease levels of Shank protein, resulting in aberrant synaptic structure. The loss of Pin1 activity, induced by oxidative stress, may also render neurons more susceptible to the toxicity of oligomers of A*β* and to excitation, thereby inhibiting NMDA receptor-mediated synaptic plasticity and exacerbating NMDA receptor-mediated synaptic degeneration. These results suggest that loss of Pin1 activity could lead to the loss of synaptic plasticity in the development of AD.

## 1. Introduction

The hallmark pathological lesions of Alzheimer's disease (AD) are *β*-amyloid (A*β*) plaques, neurofibrillary tangles, and synaptic loss [1]. Among these, A*β* plaques and tangles can be detected decades before AD symptoms arise [2–4]. Synaptic loss begins in preclinical AD and is the strongest anatomical correlate of the degree of clinical impairment [5]. The molecular pathophysiology of synaptic dysfunction remains elusive, particularly the molecular events that lead up to the loss of synaptic plasticity decades before the onset of clinical disease [6].

Oligomers of A*β*, the early aggregates of A*β* peptides, have been suggested as culprits in dysfunction of synaptic plasticity in early AD patient brains [1]. Pin1 is a unique peptidyl-prolyl isomerase that catalyzes cis-trans isomerization of phosphorylated Ser/Thr-Pro motifs. Increase in oligomers of A*β* and other age-related insults induce oxidative stress [7], which could cause the loss of Pin1 activity [8, 9]. Interestingly, loss of Pin1 facilitates formation of plaques and tangles [10–12], suppresses neuronal differentiation [13], and induces neurodegeneration [14]. The early aggregates of plaques and tangles associate with detergent-resistant rafts and with the postsynaptic density (PSD) [15, 16] which is crucial in organizing glutamate receptors within dendritic rafts. Activation of an NMDA receptor can induce the phosphorylation of several hundred PSD proteins [17]. This includes hundreds of Ser/Thr-Pro motifs [18], a set of which upon cis-trans isomerization by Pin1 may affect ubiquitin modification of proteins [19, 20]. The activity of the ubiquitin proteasome system (UPS) can directly alter the plasticity of the PSD [21, 22]. PSD proteins are organized by Shank proteins [23, 24]. Mutation of Shank3 leads to modification of ubiquitin in Shank3 protein and results in loss of glutamate receptors within an aberrant PSD structure [25–27]. Shank3 protein is lost and highly modified by ubiquitin in synapses of AD patient brains [28, 29]. Pin1 controls protein synthesis in dendritic spines [30]. These findings indicate that loss of Pin1 activity could directly affect synaptic plasticity in the brains of AD patients.

We localized Pin1 to dendritic rafts and to the PSD and found a pathological loss of Pin1 within the synapses of AD patient brains. Loss of Pin1 activity may increase the modification of ubiquitin in PSD proteins and lead to the loss of Shank protein, resulting in aberrant PSD structure. This renders the synapse more susceptible to the toxic assault of oligomers of A*β* and excitation, thereby inhibiting synaptic plasticity and inducing synaptic degeneration, which may accelerate synaptic loss in preclinical AD. These findings could distinguish Pin1 as a target with the potential to protect synaptic function in preclinical AD.

## 2. Results

### 2.1. Pin1, Phosphorylated Tau, Oligomers of A*β*, and Glutamate Receptor Coincidently Exist in Detergent-Resistant Dendritic Rafts and PSD Fractions

**Figure 1:**
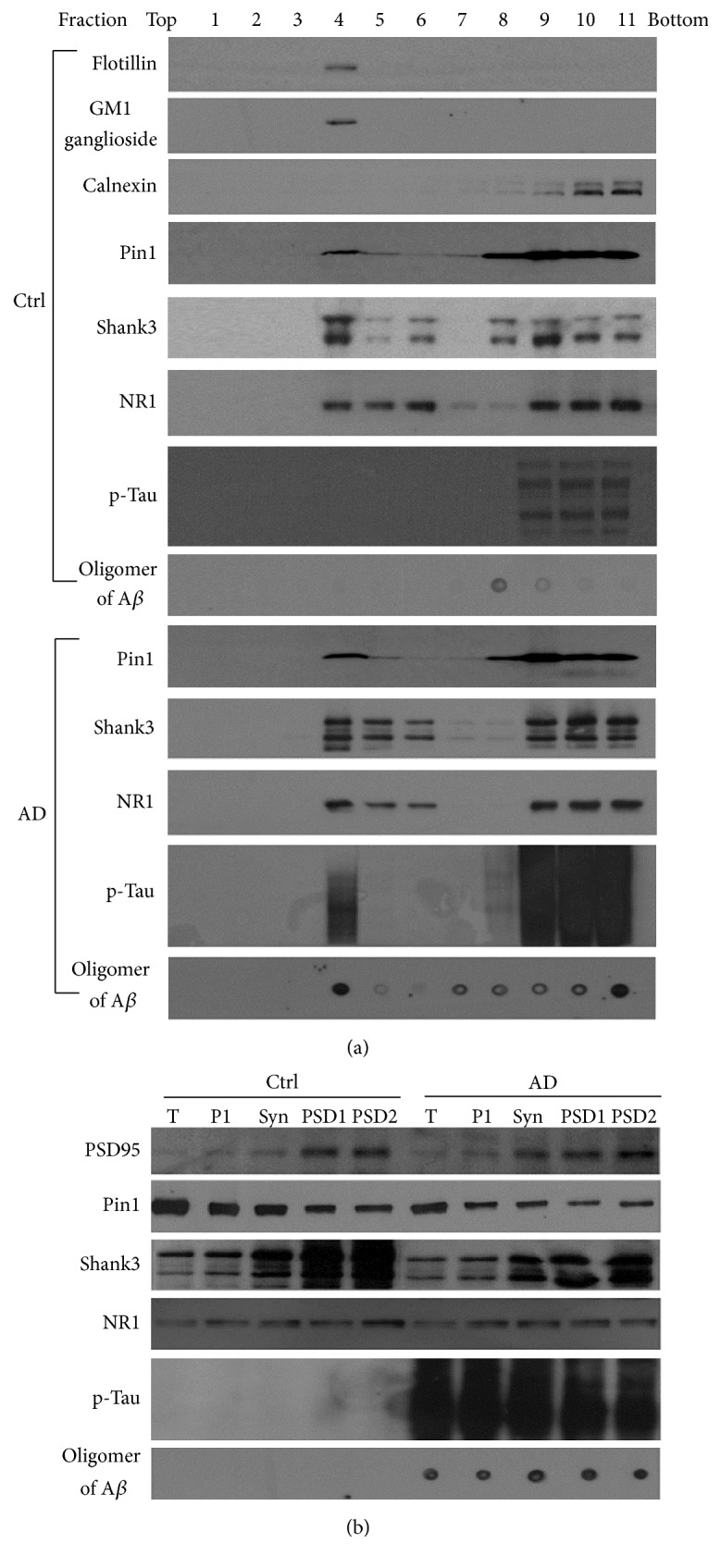
Pin1 proteins exist in detergent-resistant dendritic rafts and PSD fractions coincidently containing phosphorylated Tau, oligomers of A*β*, and glutamate receptor. The detergent-resistant synaptosome suspension was fractionated by sucrose gradient ultracentrifugation to isolate dendritic rafts and PSDs. (a) Analysis of dendritic rafts. Proteins in ultracentrifuged fractions at equal volume were analyzed by Western blot or dot blot. Fraction 4 contained flotillin and GM1 ganglioside, known markers for rafts, along with Pin1, p-Tau, oligomers of A*β*, Shank3, and NR1 proteins. Fraction 4 did not show calnexin, a marker of endoplasmic reticulum (ER). (b) Analysis of the PSD. PSD proteins at equal amounts were analyzed by Western blot or dot blot. PSD95 and Shank3, both known protein markers of the PSD, were enriched in the PSD fraction (PSD2), along with Pin1, NR1, p-Tau, and oligomers of A*β*. Ctrl: human control tissues; AD: human AD tissues; T: total tissue; P1: total membrane; NR1: a subunit of NMDA receptor; Syn: synaptosome; PSD1: pellets from the synaptosome treated once by 0.5% Triton X-100; PSD2: pellets from PSD1 treated once more by 0.5% Triton X-100.

To detect Pin1 proteins in detergent-resistant dendritic rafts and PSD, synaptosome fractions were effectively isolated from the frontal cortical tissues of human AD and normal control brains, and the synaptic markers PSD95 and Shank3 were enriched up to 9 and 8 times in synaptosome fractions, respectively (Figure 1(b)). The synaptosomes were further treated to isolate dendritic rafts and PSDs, and dendritic rafts and PSDs were analyzed by Western blot or dot blot.

First, to analyze the dendritic rafts, flotillin and GM1 ganglioside were used as markers. Both raft markers were detected in fraction 4 of the sucrose gradient. Calnexin, an ER marker used as a negative marker, was not detected in fraction 4. Pin1, NR1, and Shank3 coincidently were found in fraction 4 of the sucrose gradient from cortical tissues of human control and AD brains (Figure 1(a)). Meanwhile, phosphorylated Tau and oligomers of A*β* were detected obviously in dendritic rafts isolated from cortical tissues of human AD brains, but much less from cortical tissues of control brains (Figure 1(a)).

Second, to analyze PSD, PSD95 and Shank3 were used as markers of PSD. The PSD1 fraction was comprised of pellets of synaptosomes that were treated once by Triton X-100, and the PSD2 fraction was comprised of pellets of PSD1 treated by Triton X-100 one additional time [31] (Figure 1(b)). We found Pin1 proteins in the synaptosome and in the PSD fractions from cortical tissues of AD and control brains. Pin1 proteins exist in PSD fraction coincidently containing: PSD95, Shank3, and NR1. However, oligomers of A*β* and hyperphosphorylated Tau were observed only in PSD fractions of AD brain cortical tissues and not in PSD of control cortical tissues (Figure 1(b)). Although Pin1 proteins were not enriched in synaptosome and PSD fractions, these results suggest that Pin1 exist in dendritic rafts and in PSD.

**Figure 2:**
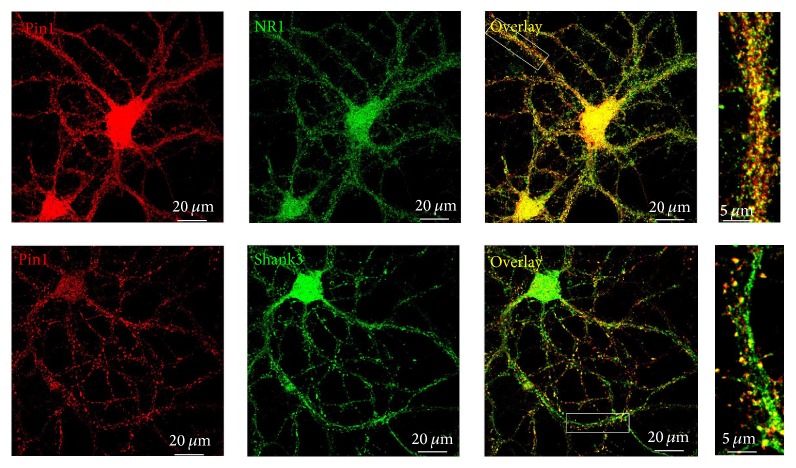
Pin1 proteins are partly colocalized with Shank3 and NR1 proteins at dendritic spines of neurons. Cortical neurons at 21 DIV were double immunolabeled for Pin1 (red) and Shank3 (green) or NR1 (green). When the images were merged, the yellow in the overlay showed colocalization of Pin1-immunoreactive puncta with Shank3 and NR1 proteins. Right panels were high-magnification images of selected overlays.

Third, to further clarify the location of Pin1 at the synapse, the cultured cortical neurons of C57/BL6 mice at 21 DIV were used to detect the distribution of Pin1, Shank3, and NR1 proteins by immunocytochemical analysis. Shank3 proteins are expressed at the PSD in the dendritic spine of excitatory neurons [23]. We found that Pin1 proteins also partly colocalized with NR1 proteins along with Shank3 proteins at the dendritic spines of excitatory neurons (Figure 2).

### 2.2. Pin1 Proteins Are Altered at Synapses of Human AD and Tg2576 AD Mice Brains

**Figure 3:**
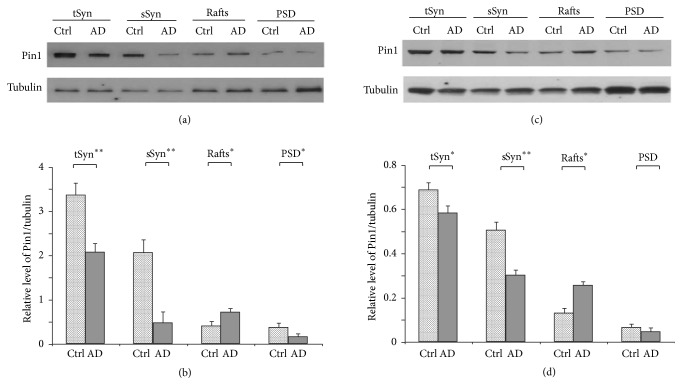
Pin1 proteins show pathological loss in the synapses of human AD and Tg2576 AD model mice. The Pin1 proteins in synaptosomes, soluble synaptosomes, dendritic rafts, and PSD fractions were isolated from human frontal cortical tissues (a and b) and from Tg2576 AD model mice cortical tissues (c and d) and were detected by Western blot. (a and c) Representative Western blot experiment. (b and d) Results from densitometric imaging of these same samples (human AD frontal cortical tissues, *n* = 8; human frontal cortical tissues, *n* = 5; Tg2576 mice, *n* = 7; and wild-type mice, *n* = 7; ^*∗*^*p* < 0.05, ^*∗∗*^*p* < 0.01). Ctrl: human control frontal cortical tissues; AD: human AD frontal cortical tissues; tSyn: the supernatant of synaptosomes extracted by 1% SDS; sSyn: the supernatant of synaptosomes extracted by 0.5% Triton X-100.

Pin1 proteins have been identified at detergent-resistant synaptic structures: dendritic rafts and PSD (Figures 1 and 2). The loss of Pin1 is involved in the formation of A*β* plaques and hyperphosphorylated Tau tangles in AD patient brains [10, 11]. To detect whether Pin1 proteins were involved in the pathological changes found in synapses of AD patient brains, the cortical frontal tissues of human AD and age-matched control brains were used to isolate synaptosomes (Figure 1(b)). Synaptosomes were fractioned in 0.5% Triton X-100 by ultracentrifuge into a soluble synaptic fraction, synaptic rafts fraction, and PSD fraction. The Pin1 proteins in these fractions were separated by 16.5% Tris-tricine gel and the levels of these Pin1 proteins were detected by Western blot. We found total synaptic Pin1 protein to be significantly lost by 39% in human AD patient frontal cortical tissues compared with that in control tissues. The soluble synaptic Pin1 proteins were also significantly decreased by 76% in these AD samples, and the detergent-resistant Pin1 was significantly increased in dendritic rafts but significantly decreased in PSD fractions from cortical tissues of AD patient brains (Figure 3(a)). To confirm these pathological changes, the experiment was repeated with cortical tissues isolated from cortical tissues of Tg2576 amyloid-AD model mice brains at 18 months, which are equivalent to 55 years in human [32]. The total synaptic Pin1 protein and soluble synaptic Pin1 protein also showed significant loss, and the levels of Pin1 protein at dendritic rafts showed significant increase in Tg2576 mice brains (Figure 3(b)). These results indicate that the pathological loss of Pin1 proteins at the synapse could be involved in synaptic dysfunction in AD development.

### 2.3. Blocking of Pin1 Activity Increases the Modification of Ubiquitin in PSD Proteins

**Figure 4:**
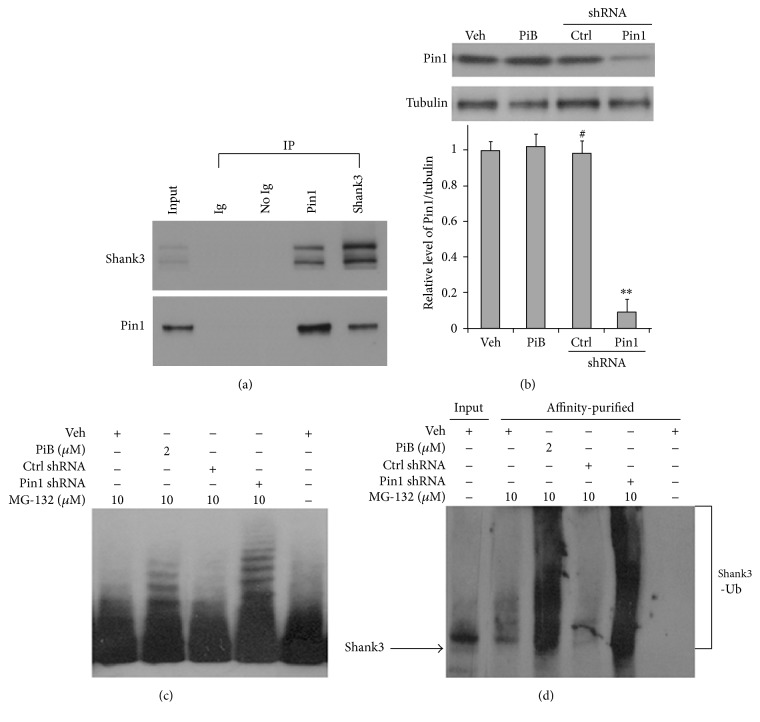
Blocking of Pin1 activity increases the modification of ubiquitin in PSD proteins. C57/BL6 cortical neurons at 21 DIV were incubated with PiB and MG-132 for 24 h and transfected by Pin1 shRNA or control shRNA lentivirus for 48 h, respectively; the PSDs were isolated and analyzed with Western blot. (a) Coimmunoprecipitation between Pin1 and Shank3 proteins. (b) Total Pin1 proteins were knocked down (*n* = 7 dishes for each experimental condition, ^*∗∗*^*p* < 0.01; ^#^*p* > 0.05). (c) Ladders of Shank3 proteins after the loss of Pin1 activity. (d) Ubiquitinated Shank3 proteins were enriched by ubiquitin-affinity purification and recognized by Shank3 antibody in Western blot.

The activity of Pin1 protein affects ubiquitin modification of proteins [20], and the activity of the UPS can directly alter the plasticity of the PSD [21, 22]. The loss of Pin1 could alter the modification of ubiquitin in PSD proteins, leading to the alteration of structural and functional plasticity of the PSD. To test this hypothesis, cultured cortical neurons of C57/BL6 mice at 21 DIV were incubated with PiB, the selective inhibitor of Pin1 [33, 34], and MG-132, a proteasome inhibitor, for 24 h at different concentrations (Figure 4(a)). The neurons were collected and PSD fractions were isolated. PSD proteins at equal amounts were analyzed by Western blot. The ubiquitin-labeled PSD proteins were detected by anti-ubiquitin antibodies. Ubiquitin-labeled PSD proteins significantly increased after the cultured neurons were incubated with PiB for 24 h (Figures 4(c) and 4(d)). Because Shank3 protein is a core protein of PSD [23] and Shank3 protein shows polyubiquitination and loss in AD [28, 29], Shank3 protein was analyzed in this test. We observed Pin1 protein and Shank3 protein in synaptosome from cortical tissues of human control brains showed associated together by immunoprecipitation (Figure 4(a)). Meanwhile, Pin1 shRNA was used to knock down Pin1 protein in cultured neurons; the level of Pin1 protein was decreased by about 82% after neurons were transfected by Pin1 shRNA lentivirus for 48 h (Figure 4(b)). We used these test conditions to treat cultured neurons with PiB for 24 h and with Pin1 shRNA for 48 h. The PSD fractions were then isolated. When the amount of PSD protein was increased to 75 *μ*g for Shank3 Western blot, ladders of Shank3 proteins were apparent in the PSD fractions after treatment with Pin1 inhibitor PiB or Pin1 shRNA (Figure 4(c)). To confirm that these ladders of Shank3 proteins contained ubiquitin modification, we enriched the ubiquitinated proteins in isolated PSD using a polyubiquitin enrichment kit. These ubiquitinated proteins were recognized by Shank3 antibody in Western blot, and Shank3 showed obvious ubiquitination (Figure 4(d)). These results indicate that loss of Pin1 activity may lead to an increase in ubiquitinated proteins in the PSD, which could increase the degradation of Shank3 and other PSD proteins [28, 29], leading to changes in the structure of the PSD in AD development.

### 2.4. Blocking of Pin1 Activity Disrupts the Structural Plasticity of PSD

**Figure 5:**
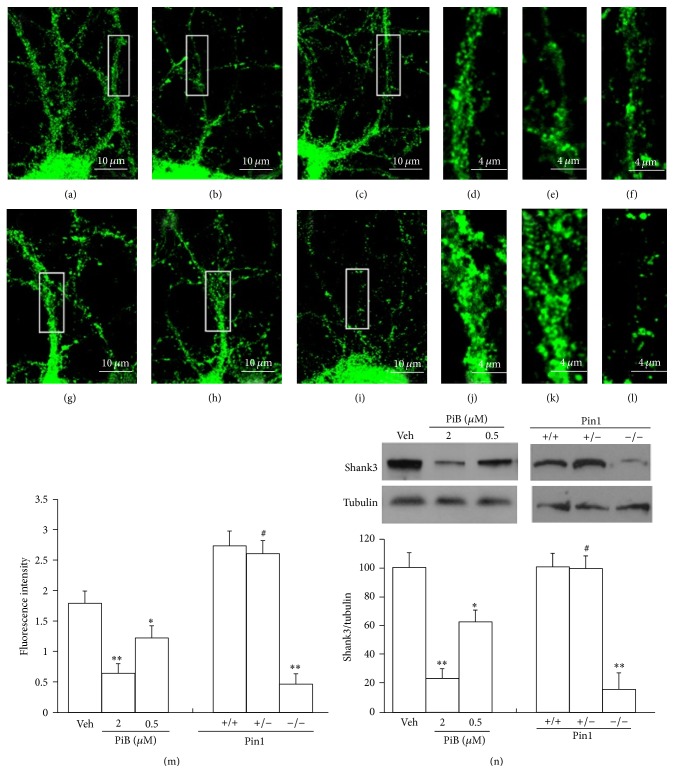
Blocking of Pin1 activity leads to degradation of Shank3 proteins in PSD. Cultured cortical neurons at 21 DIV were prepared on coverslips for immunofluorescence detection and in 10 cm dishes for immunoblot assay. Panels (a)–(f) showed the cultured cortical neurons of C57/BL6 mice at 21 DIV treated with vehicle (a and d), 2 *μ*M PiB (b and e), or 0.5 *μ*M PiB (c and f) for 24 h; panels (d), (e), and (f) were the high-magnification images of selected fields. Panels (g)–(l) showed the cultured cortical neurons of Pin1^+/+^ (g and j), Pin1^+/−^ (h and k), and Pin1^−/−^ (i and l) mice at 21 DIV; panels (j), (k), and (l) were the high-magnification images of selected fields. These neurons on coverslips were fixed, permeabilized, and labeled by Shank3 antibody for immunofluorescence detection by immunocytochemistry. (m) Immunofluorescence intensity demonstrated a large PiB-induced decrease in Shank3 proteins from seven independent experiments (~90 neurons for each experimental condition, ^*∗∗*^*p* < 0.01; ^*∗*^*p* < 0.05). (n) Western blots showed significant decrease in the levels of Shank3 proteins in PiB-treated or in Pin1^−/−^ neurons compared with vehicle-treated or Pin1^+/+^ neuron extracts, respectively (*n* = 7 dishes for each experimental condition, ^*∗∗*^*p* < 0.01; ^*∗*^*p* < 0.05; ^#^*p* > 0.05).

Thousands of proteins interact together at the PSD. Shank proteins organize these proteins to form macromolecules including NMDA, AMPA, and mGlu receptors [23]. Because of Shank proteins' large organizational role, the loss of Shank3 proteins and the extensive modification of ubiquitin in Shank3 proteins may result in aberrant PSD structure and glutamate receptor loss [26, 27]. Shank3 proteins show loss and are highly modified by ubiquitin in AD [28, 29]. To evaluate the pathological degradation of PSD proteins after loss of Pin1 activity in AD brain, we selected Shank3 as the functional and structural marker of changes in PSD. The mature synapses at 21 DIV are more sensitive to NMDA and other insults. Cultured neurons of C57/BL6 mice at 21 DIV were treated by PiB at 0.5 *μ*M and 2.0 *μ*M to block Pin1 activity and with control vehicle for 24 h (Figures 5(a)–5(f)), while cultured Pin1^+/+^, Pin1^+/−^, and Pin1^−/−^ neurons at 21 DIV were also examined (Figures 5(g)–5(l)). The neurons on coverslips were analyzed using immunocytochemistry to detect Shank3 protein levels, and the neurons in dishes were used to isolate PSD fractions, which were analyzed by Western blot. We found that C57/BL6 neurons treated with PiB showed significant reduction in Shank3 protein levels (Figures 5(a)–5(f), 5(m), and 5(n)). These results indicated that the loss of Pin1 activity could lead to the loss of Shank3 protein and thus affect the structure of the PSD. The same results were replicated in cultured Pin1^−/−^ neurons, but not in Pin1^+/+^ or Pin1^+/−^ neurons at 21 DIV (Figures 5(g)–5(l), 5(m), and 5(n)). To avoid synaptic degeneration due to the increased A*β* peptide after the loss of Pin1 activity [11], we used cultured cortical neurons from *β*-amyloid precursor protein (APP)^−/−^ mice to repeat these experiments. The inhibitor of Pin1 and the Pin1 shRNA can also decrease the levels of Shank3 proteins in neurons of APP^−/−^ mice in vitro (data not shown). These results suggest the role of loss of Pin1 activity in reducing levels of Shank3 protein, in addition to direct A*β* peptide toxicity in cultured neurons.

### 2.5. Inhibition of Pin1 Activity Blocks NMDA Receptor-Mediated Turnover of Shank3 and PSD95 Proteins and Increases NMDA Receptor- and A*β* Oligomer-Mediated Degradation of Shank3 and PSD95 Proteins

**Figure 6:**
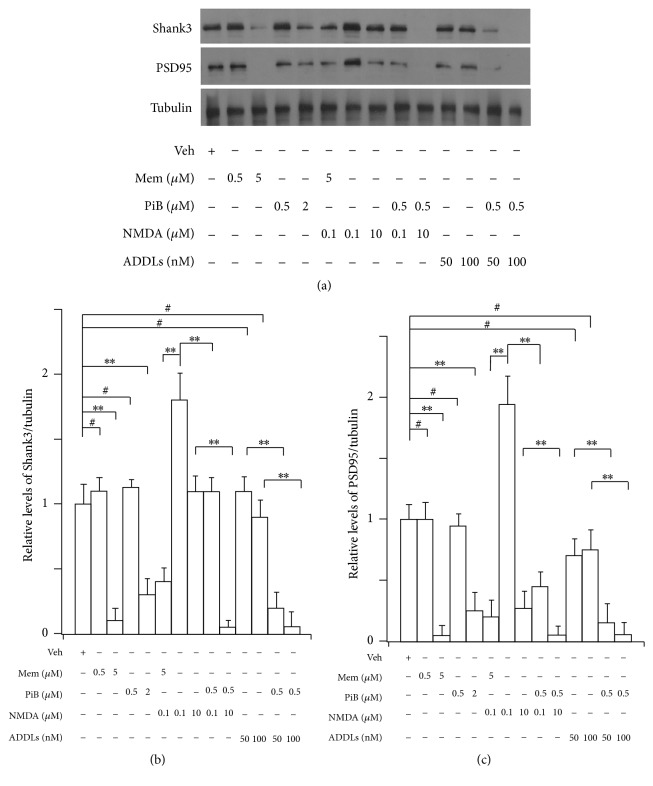
Blocking of Pin1 activity blocks NMDA receptor-mediated turnover of Skank3 and increases degradation of Shank3 and PSD95 induced by oligomers of A*β* and NMDA in cultured neurons. Cortical neurons at 15 DIV were incubated with memantine, NMDA, PiB, and oligomers of A*β* for 24 h. (a) Representative Western blot. (b and c) Results from densitometric imaging of these tests. Memantine 10 *μ*M significantly decreased levels of Shank3 and PSD95 proteins. NMDA 100 nM significantly increased levels of these proteins, and 10 *μ*M memantine significantly inhibited the increase of Shank3 induced by 100 nM NMDA. When 10 *μ*M NMDA and 0.5 *μ*M PiB were incubated with neuron together, these proteins were significantly decreased. The same results were observed between PiB and oligomers of A*β* from seven independent experiments (*n* = 7 dishes for each experimental condition, ^*∗∗*^*p* < 0.001, ^#^*p* > 0.05).

The activity of synaptic NMDA receptors regulates the dynamics of proteins in PSD including Shank3 and PSD95 protein [19, 35]. Because the synapses of cortical neurons are functional at 12 DIV and mature at 21 DIV [36] and Pin1 protein is already colocalized with NR1 and Shank3 proteins at dendritic spine at 15 DIV (data not shown), to detect how the loss of Pin1 activity affects levels of Shank and PSD95 proteins in neurons, here we used cortical neurons at 15 DIV to observe NMDA receptor-mediated turnover of Shank3 and PSD95 proteins after the loss of Pin1 activity. Memantine, a noncompetitive antagonist of NMDA receptors, has been used clinically in the treatment of AD to block neuronal oxidative stress [7]. In this test, we selected memantine and another selective competitive antagonist of NMDA receptor AP-5 to block NMDA receptor and observe the effects of Pin1 on levels of Shank3 and PSD95 proteins induced by NMDA at 0.1 *μ*M and 10 *μ*M. According to several rodent studies and human clinical trials, at 10 *μ*M memantine exerts its effect at NMDA receptors [37–39] in addition to other receptors. We selected 0.5 and 5 *μ*M concentrations of memantine for this test. After memantine, NMDA, PiB, and oligomers of A*β* were added to cultured neurons for 24 h (Figure 6), neurons were then collected, and levels of Shank3 and PSD95 proteins were detected by Western blot. 5 *μ*M memantine significantly decreased levels of Shank3 and PSD95 proteins. 50 nM (data not shown) and 100 nM NMDA significantly increased the levels of Shank3 and PSD95 proteins; 5 *μ*M memantine (Figure 6) and 0.5 *μ*M AP-5, a selective antagonist of NMDA receptor (data not shown), blocked the increase of Shank3 and PSD95 proteins in neurons induced by 100 nM NMDA (Figure 6). These results suggest the ability of 50 nM and 100 nM NMDA to induce an increase in Shank3 and PSD95 proteins and to protect synaptic function [36, 40, 41]. After the loss of Pin1 activity in the presence of PiB, the NMDA receptor-mediated increase of Shank3 and PSD95 proteins was significantly blocked (Figure 6). Meanwhile, we found that 10 *μ*M NMDA or 0.5 *μ*M PiB individually did not change the level of Shank3 protein; however when both were incubated with cultured neurons together, levels of Shank3 protein were significantly reduced. 0.5 *μ*M PiB also increased the reduction of Shank3 and PSD95 proteins induced by oligomers of A*β* (Figure 6). These findings indicate that the loss of Pin1 activity could block NMDA receptor-mediated turnover of Shank3 and PSD95 proteins and increase NMDA receptor- and A*β* oligomer-mediated degradation of Shank3 and PSD95 proteins, contributing to synaptic loss in AD development.

## 3. Discussion

Pin1 protein regulates the function of mitotic phosphoproteins and determines cell-cycle progression [9]. The loss of Pin1 is a common pathological cause linked to the production of A*β* and hyperphosphorylated Tau in AD [10, 11, 42]. Here we identified and showed the association of Pin1 with Shank proteins at dendritic rafts and the PSD, in which both fractions are associated with NR1; this finding suggests Pin1 may regulate signal transduction at dendritic rafts and signal processing at the PSD. The loss of Pin1 activity alters the modification of ubiquitin in PSD proteins and leads to the loss of Shank3 protein. As a result, Pin1 loss may make aberrant synapses more susceptible to the toxic effects of molecules such as oligomers of A*β* and glutamate, thereby inhibiting NMDA receptor-mediated turnover of Shank protein and synaptic generation, and exaggerating NMDA receptor-mediated synaptic degeneration. Pin1 could play a pathological role in synaptic dysfunction in addition to the formation of misfolded proteins in preclinical stages of AD.

With aging, the activity of Pin1 protein may be lost by oxidation in mild cognitive impairment [8, 43], which could lead to the formation of A*β* plaques and hyperphosphorylated Tau in preclinical stages of AD [10–12]. We observed that the levels of Pin1 proteins are significantly lost in the synaptic fractions of AD brain cortical tissues (Figure 3), in addition to the loss of Pin1 protein in total AD brain tissues [10]. In vitro, the loss of Pin1 activity increases the modification of ubiquitination in PSD proteins (Figure 4). Modification of synaptic proteins mediates the protein-protein interactions which are required for structural plasticity of excitatory synapses. The loss of Pin1 activity may be one of the earliest events leading to the pathological modification of synaptic proteins in preclinical AD (Figure 4) [42, 43].

Synaptic NMDA receptors protect synaptic function and stimulate synaptic generation to maintain homeostasis [36, 40] (Figure 6). Pin1 protein is found in dendritic spines and regulates the synthesis of PSD 95 [30]. Here we found that Pin1 proteins associate with dendritic rafts and the PSD fractions containing NMDA receptors and Shank3 proteins (Figure 1), which suggests that Pin1 may regulate signal transduction at dendritic rafts and may influence the function of Shank3 proteins and other proteins within dendritic rafts and the PSD. The normal level of glutamate in brains is 1–4 *μ*M [44]. The baseline concentration of extracellular glutamate is also reported to be near 25 nM [45]. We selected various concentrations of NMDA to regulate the structural changes in the PSD. Shank proteins have the capability to turn over within minutes [35], organize other scaffolding proteins in addition to glutamate receptors, and induce spine formation [41]. Conversely, the loss of Shank3 protein can lead to aberrant structure of the PSD and loss of NMDA receptors [27]. In our results, 50 nM (data not shown) and 100 nM NMDA increased Shank3 protein levels (Figure 6). Under these conditions, NMDA may protect synapses and increase synaptic formation [36, 40]. On the other hand, memantine and AP-5 blocked the increase of Shank3 proteins induced by NMDA at 100 nM. PiB, the inhibitor of Pin1, also clearly blocked elevations in Shank3 proteins induced by NMDA at 100 nM, which implicates the blocking of Pin1 activity in inhibiting the synthesis of Shank3 induced by NMDA receptor. This confirms that Pin1 may control protein synthesis in dendritic spines [30]. Interestingly, we also found that the inhibitor of Pin1 could increase NMDA receptor-mediated excitotoxicity, leading to the degradation of Shank3 proteins in cultured neurons (Figure 6), which suggests that the loss of Pin1 activity can increase NMDA receptor-mediated excitotoxicity in dendritic spines. To avoid the effects of increased A*β* peptides on the synapse after the loss of Pin1 activity, we used cultured cortical neurons of APP^−/−^ mice and found that PiB or Pin1 shRNA was also capable of inducing Shank3 loss. These results suggest that Pin1 may play a role in the NMDA receptor-mediated functional and structural plasticity of the PSD, and the loss of Pin1 activity may block normal NMDA receptor-mediated synaptic formation thereby increasing the neuron's susceptibility to oligomers of A*β* or other toxic events such as excitation (Figure 6). During this pathological process, the accumulation of modifications at the excitatory synapse may lead to an irreversible synaptic dysfunction and aberration of synaptic structure that cannot be normalized by synaptic NMDA receptors [36, 40], ultimately accelerating synaptic loss.

The PSD is a multiprotein complex organized by Shank proteins [23, 24]. Mutations in this set of proteins are involved in over 133 genetic neurological diseases [24] including Shank-relevant diseases [27]. The loss of Pin1 activity could affect conformational and functional changes in PSD proteins in AD and in other neurological diseases. Pin1 activity often translates to a fate-determining ubiquitination switch, and Pin1 may likewise affect the degree of ubiquitination in the degradation of PSD proteins (Figure 4) including PSD95, GKAP, Shank, and other proteins involved in the disruption of the PSD in AD [21, 28, 46, 47]. Shank3 and other scaffolding proteins, PSD95 and PSD93, undergo proline-directed phosphorylation and share a similar phosphorylation motif [48]. The alteration of Shank protein levels and modification of ubiquitin have been linked to AD [28, 29]. The loss of Pin1 could affect the modification of proteins in the PSD, leading to the degradation of proteins by the UPS (Figure 4) and contributing to the aberrant structure of the PSD in AD. Proteasome activity declines with aging and in AD brains [49], and we found that loss of Pin1 activity can significantly increase the ubiquitin modification and degradation of Shank3 protein in cultured neurons (Figures 4, 5, and 6). These results suggest that the NMDA receptor-mediated activity of the ubiquitin system can be altered, leading to the degradation of PSD proteins after the loss of Pin1 activity in dendritic spine. In addition, Pin1 could also directly bind to phosphorylated Shank3 protein and affect the levels of Shank3 protein in dendritic spine. These relevant molecular mechanisms warrant further investigation.

## 4. Experimental Procedure

### 4.1. Human AD and Mice Brain Tissues

Human brain tissues were obtained at autopsy from 8 patients diagnosed clinically and histopathologically with AD (80.9 ± 2.7 years) in the postmortem period (5.5 ± 0.8 h) and from 5 age-matched controls with no clinical or morphologic evidence of brain pathology (81.4 ± 2.0 years) in the postmortem period (5.0 ± 1.0 h). The ages and PMDs of cases were not significantly different between the AD and control group. In this study, we focused on excitatory synapses and the potential effects of medications on glutamate receptor levels. None of our control or AD subjects were on memantine, an antagonist of NMDA receptor, or other NMDA receptor modulators. All tissues were obtained from the DUCOM Memory Disorders brain bank.

Pin1 knockout mice were bred in Flavio Rizzolio's laboratory (Temple University, Philadelphia, PA). Tg2576 mice were bred in the animal facility center at Drexel University College of Medicine.

### 4.2. Cortical Neuron Culture and Treatment

Cortical neurons of C57/BL6 mice at E18 and Pin1 knockout mice at P0 were cultured on coverslips for immunocytochemical analysis, in 10 cm dishes for dendritic raft or PSD isolation or in 24-well plates for Western blot, as described previously [50, 51]. To culture the neurons of Pin1^−/−^ mice, the cortical neurons of pups at P0 from Pin1^+/−^ mice cross-bred with Pin1^+/−^ mice were cultured individually, and the pups were genotyped. These cultured cortical cells at various times (from 12 to 21 DIV for different tests) were treated with memantine, AP-5, NMDA, PiB, Pin1 shRNA lentivirus particle, control shRNA lentivirus particle, and vehicle as indicated in the figures. PiB, a selective Pin inhibitor [33, 34], was used to block the activity of Pin1. Pin1 shRNA lentivirus particles were used to create Pin1 knockdowns [52].

### 4.3. Synaptosome, Dendritic Raft, and PSD Preparation

Synaptosomes were prepared from frontal cortical tissues of human brains, from cultured neurons, or from cortical or hippocampal tissues of mice brains [28, 53]. Dendritic rafts fractions were prepared from synaptosomes [15, 54]. PSD fractions were prepared from synaptosomes [31].

### 4.4. Ubiquitinated-Protein Affinity Isolation

PSD fractions were isolated from cultured neurons with or without treatment of PiB, Pin1 shRNA lentivirus particles, control shRNA lentivirus particles, or vehicle. Proteins from these PSD fractions at equal amounts were solubilized in TBS buffer (10 mM Tris-HCl, 100 mM NaCl) with 0.1% SDS at 70°C for 15 min. The ubiquitinated proteins in supernatant at 100,000 ×g for 30 min were pulled down by ubiquitin-interacting motif affinity gel (polyubiquitin enrichment kit, Pierce Biotechnology) according to the manufacturer's instructions. These gels were washed three times by TBS buffer with 0.1% SDS. Finally, 1% SDS Laemmli sample buffer was used to elute ubiquitinated proteins for Western blot analysis.

### 4.5. Oligomers of A*β* (ADDLs) Preparation

Oligomers of A*β*_1–42_ peptide were prepared as previously described [50, 55].

### 4.6. Immunoblot

Western and dot blot analyses were based on published procedures [50]. For Western blot analysis, the samples were separated by 4%–20% glycine-HCl SDS-polyacrylamide gel or 16.5% Tris-tricine SDS-polyacrylamide gel electrophoresis and then transferred to nitrocellulose membrane. For dot blot analysis, the samples of dendritic rafts, PSDs, and synaptosome fractions were treated by 0.1% SDS or by Ham's F-12 medium, and proteins of these samples in equal amounts were dotted on nitrocellulose membrane. The proteins were recognized by specific antibodies and visualized with ECL. Antibodies to flotillin (rabbit), Shank3 (rabbit and goat), NR1 of NMDA receptor subunit (rabbit), PSD95 (goat), Pin1 (mouse and rabbit), calnexin (rabbit), and *β*-tubulin (rabbit) were from Santa Cruz Biotechnology (Santa Cruz, CA). Antibody A11 to oligomers of A*β* was from Invitrogen [56]. Antibody (rabbit) to Tau [p-231] was from Covance (Emeryville, CA). A polyubiquitin enrichment kit and antibody to ubiquitin (rabbit) were obtained from Pierce, and Alexa-conjugated secondary antibodies were from Invitrogen. Hybond ECL nitrocellulose, HRP-conjugated secondary, and rainbow protein ladders were from Amersham Pharmacia Biosciences.

### 4.7. Coimmunoprecipitation

IP was carried out by incubation of synapse lysates of human control brain cortical tissues. Synaptosomes containing 200 *μ*g protein were resuspended in buffer A containing 50 mM Tris (pH 7.5), 100 mM NaCl, 1.5 mM EGTA, 0.1% SDS, and 1% Triton X-100 for 1 hour at 4°C. The supernatant from 100,000*g* for 30 minutes was incubated with specific antibodies plus Protein G agarose (Invitrogen) at 4°C overnight, followed by washing 5 times in a buffer B containing 50 mM Tris (pH 7.5), 100 mM NaCl, and 1.5 mM 0.1% Triton X-100. Antibodies used for IP were rabbit polyclonal anti-Pin1 and anti-Shank3 (Santa Cruz) and rabbit IgG (Sigma-Aldrich) as negative controls. The IP was analyzed by Western blot using mouse monoclonal anti-Pin1 and goat polyclonal anti-Shank3 (Santa Cruz).

### 4.8. Immunocytochemistry

Treated cells were rinsed with neurobasal media and then fixed with 3.7% formaldehyde in neurobasal media (1 : 1 volume) for 20 min followed by an additional 20 min of undiluted fixative. Cells were rinsed extensively in PBS. Coverslips were incubated in blocking solution (10% BSA in PBS with or without 0.1% Triton X-100) for 45 min at room temperature. Antibodies were used against Pin1, NR1, and Shank3. Primary antibodies were diluted in blocking solution and incubated overnight at 4°C. After rinsing with PBS with 1% BSA, coverslips were incubated with appropriate Alexa-conjugated secondary antibodies diluted in PBS plus 1% PBS for 90 min at room temperature, rinsed in PBS three times, and mounted with ProLong Antifade media [51]. Quantitative analysis of the immunofluorescence intensity at dendritic arbors was performed by histogram analysis using ImageJ. Shank3 proteins were expressed in excitatory neurons and at the PSDs of dendritic spines [23]. Cell bodies were digitally removed from images; the Shank3 proteins at dendritic arbors were analyzed. Thirty images were acquired under each experimental condition, and these tests were done in triplicate. The data were pooled for quantitative estimates of changes in Shank3 protein. Three independent experiments were performed for each test [7].

### 4.9. Statistical Analysis

For each experiment, two or three independent replicated experiments were performed. The densities of immunoblot were acquired with densitometric scan and quantified with ImageJ. Results were expressed as means ± SEM. The data were analyzed with one-way analysis of variance. Statistical significance was determined at *p* < 0.05.

## 5. Conclusion

Here we found Pin1 proteins exist in detergent-resistant dendritic rafts and PSDs at the precise location of key macromolecules including NMDA, AMPA, mGlu receptors, and Shank proteins for synaptic plasticity. The loss of synaptic Pin1 activity may alter the modifications of ubiquitin in PSD proteins and lead to the loss of Shank3 proteins and formation of aberrant synapses which are more susceptible to toxic effects of molecules such as oligomers of A*β* and glutamate. Toxicity thereby inhibits NMDA receptor-mediated turnover of Shank3 and PSD95 and exaggerates NMDA receptor-mediated loss of Shank3 and PSD95 proteins. These multiple factors could work together to exacerbate synaptic dysfunction in preclinical AD. The loss of Pin1 activity could play an integral role in the pathogenesis of synaptic dysfunction contributing to the onset of clinical AD.

## Abbreviations

AD: Alzheimer's disease
Pin1: Peptidyl-prolyl cis-trans isomerase NIMA-interacting 1
PSD: Postsynaptic density
NR1: N-Methyl-D-aspartate (NMDA) receptor 1
UPS: Ubiquitin proteasome system
A*β*: *β*-Amyloid peptides
ADDLs: A*β* oligomeric ligands
PiB: Benzo[lmn][3,8]phenanthroline-2,7-diacetic acid, 1,3,6,8-tetrahydro-1,3,6,8-tetraoxo-, 2,7-diethyl ester, Pin1 selective inhibitor
MG-132: Z-Leu-Leu-Leu-al, proteasome inhibitor.
